# Infection Prevention and Control Knowledge, Attitudes, and Practices of Healthcare Workers in Tertiary Care Hospitals in Bangladesh During COVID-19: A Multicenter Cross-sectional Survey

**DOI:** 10.1093/cid/ciaf246

**Published:** 2025-06-25

**Authors:** Md Golam Dostogir Harun, Shariful Amin Sumon, Md Mahabub Ul Anwar, Tahrima Mohsin Mohona, Aninda Rahman, Syed Abul Hassan Md Abdullah, Md Saiful Islam, Lisa P Oakley, Paul Malpiedi, Ashley R Styczynski, S Cornelia Kaydos-Daniels

**Affiliations:** Programme for Emerging Infections, Infectious Diseases Division, icddr,b, Dhaka, Bangladesh; Programme for Emerging Infections, Infectious Diseases Division, icddr,b, Dhaka, Bangladesh; Office of Oral Health, Maryland Department of Health, Baltimore, Maryland, USA; Programme for Emerging Infections, Infectious Diseases Division, icddr,b, Dhaka, Bangladesh; Communicable Disease Control, Directorate General of Health Services, Dhaka, Bangladesh; SafetyNet, Dhaka, Bangladesh; School of Population Health, University of New South Wales, Sydney, New South Wales, Australia; Centers for Disease Control and Prevention (CDC), Atlanta, Georgia, USA; Centers for Disease Control and Prevention (CDC), Atlanta, Georgia, USA; Centers for Disease Control and Prevention (CDC), Atlanta, Georgia, USA; Centers for Disease Control and Prevention (CDC), Atlanta, Georgia, USA

**Keywords:** IPC, KAP, HCWs, LMIC, Bangladesh

## Abstract

**Background:**

Healthcare workers (HCWs) play a pivotal role in preventing healthcare-associated infections by adhering to infection prevention and control (IPC) practices. This study assessed IPC knowledge, attitudes, and practices (KAP) among HCWs at tertiary care hospitals in Bangladesh during the coronavirus disease 2019 pandemic.

**Methods:**

From September 2020 to January 2021, semistructured questionnaires were administered to physicians, nurses, and cleaning staff at 11 tertiary care hospitals in Bangladesh. KAP components were classified into “good,” “fair,” and “poor” based on the frequency of favorable responses (>75%, 50%–75%, <50%). Multivariate logistic regression was used to assess the relationship between knowledge, attitudes, and self-reported practices.

**Results:**

We enrolled 1728 HCWs, including 526 physicians (30.4%), 934 nurses (54.1%), and 268 cleaning staff (15.5%). Physicians and nurses demonstrated “good” IPC knowledge (median 94.8% and 96.6% favorable responses, respectively) and self-reported IPC practices (median 76.2% and 80.4% favorable responses). However, most cleaning staff exhibited “poor” IPC knowledge (median 47.3% favorable responses) and practices (21.3% favorable responses). Across all categories of HCWs, the median attitude score was “fair” (range 60.0%–71.2% favorable responses). Having a positive attitude toward IPC was associated with increased IPC knowledge (adjusted odds ratio 3.0, *P* < .001) and good IPC practices (adjusted odds ratio 16.3, *P* < .001).

**Conclusions:**

HCW's KAP toward IPC was found to be suboptimal, especially among cleaning staff. However, the strong association noted between favorable attitudes toward IPC and adherence to safe IPC practices demonstrates the need for hospital leadership to promote a positive IPC culture, in addition to training and resources, to improve IPC practices and enhance healthcare resiliency beyond the coronavirus disease 2019 pandemic.

## Teaser

The study identified several specific infection prevention and control knowledge and practice gaps among healthcare workers, especially among cleaning staff, that need to be addressed through regular training, availability of resources, and demonstrating a positive culture around infection prevention and control to ensure resiliency beyond the COVID-19 pandemic.

## BACKGROUND

Infection prevention and control (IPC) is a practical and evidence-based method to avoid preventable infections in hospitals [1]. IPC practices include ensuring adequate hand hygiene, use of personal protective equipment (PPE), safe handling and disposal of sharps and waste, decontamination of medical equipment and devices, and environmental cleaning [2, 3]. Shortfalls in the implementation of IPC measures lead to a greater burden of healthcare-associated infections (HAIs) [4, 5]. The importance of IPC was highlighted during the coronavirus disease 2019 (COVID-19) pandemic when lapses in IPC contributed to the healthcare-associated spread of severe acute respiratory syndrome coronavirus 2 as well as other HAIs [6–8].

HAIs are a major public health concern, affecting millions of people annually [9, 10], and are particularly a concern in low- and middle-income countries where the risk of acquiring HAIs is 2–13 times higher than in high-income countries [11]. Reasons for higher HAI rates in low- and middle-income countries have been attributed to limited budgets, deficient or rudimentary infrastructure, a shortage of basic PPE, poor sanitary conditions, lack of good hygiene practices, understaffing, heavy workloads, a lack of IPC training, and a lack of IPC practice monitoring [12–14]. Thus, effective IPC programs within healthcare facilities are an essential cornerstone to ensuring patient safety and quality of care [15]. Yet, many institutions and healthcare workers (HCWs) face barriers to maintaining or strengthening IPC programs, which were particularly highlighted during the COVID-19 pandemic [16, 17].

Data suggest that the knowledge, attitudes, and practices (KAP) of HCWs have a profound impact on the effectiveness of IPC programs [18, 19]. Identifying effective intervention efforts to improve IPC depends on understanding existing IPC KAP among HCWs. In Bangladesh, there is a paucity of data regarding HCW's IPC KAP, but the stark number of deaths among HCWs early in the COVID-19 pandemic suggests that deficits exist [20]. This study aimed to assess the IPC KAP of physicians, nurses, and cleaning staff during the COVID-19 pandemic and to determine the relationship between IPC knowledge, attitudes, and self-reported practices at multiple tertiary care hospitals in Bangladesh. In this context, cleaning staff were grouped with other HCWs because they often assist in direct patient care, particularly in the setting of insufficient nurse staffing. The study findings aim to provide evidence for policymakers, hospital administrators, and IPC focal persons. These insights will help inform practical and resilient strategies to strengthen IPC in healthcare settings beyond the COVID-19 pandemic.

## METHODS

### Study Design and Setting

Between September 2020 and January 2021, this multicenter cross-sectional survey was conducted in 11 tertiary care hospitals across the country, including 9 public and 2 private hospitals. This convenience sample represented one-fourth of all tertiary care hospitals in the country and was selected by the Bangladesh Ministry of Health because these hospitals served as COVID-19 referral hospitals. The participating hospitals had bed capacities ranging from 450 to 2600 and varied in availability of specialty medical care.

### Study Participants and Sampling Procedure

Participants in this study included HCWs from each hospital. We enrolled physicians, nurses, and cleaning staff who were directly involved with patient care in the study hospitals. Cleaning staff were included in the study because in Bangladesh, cleaning staff are frequently involved in patient care activities including patient feeding, toileting, and transport in addition to their environmental cleaning and waste management responsibilities. Approximately 25% of HCWs were randomly selected from each group from selected hospitals with a targeted sample size ranging from 114 to 513 within each facility.

### Data Collection

We used a semistructured KAP questionnaire to obtain information on HCWs’ IPC knowledge, attitudes, and self-reported practices. The tool was developed based on literature review and expert opinion [21, 22] and was piloted at 2 facilities before study initiation. Pilot data are not included in the study data presented here. The questionnaire included 25 questions comprising 6 knowledge questions, 6 attitude questions, and 13 practice questions. The knowledge domain mainly emphasized familiarity with national healthcare IPC guidelines, standard precautions, and isolation and cohorting practices. Attitude questions focused on opinions of IPC policies and procedures, hand hygiene, and the use of various PPE. Practice-related questions centered on compliance with different IPC measures and attendance at mandatory IPC in-service trainings or workshops. The response options included “Agree,” “Disagree,” “Don't know,” or “Not applicable” for knowledge and attitude domains, and “Always,” “Often,” “Sometimes,” “Never,” or “Not applicable” for the practice domain. The duration of each face-to-face interview was approximately 30 to 35 minutes.

### Statistical Analysis

For summarizing responses and conducting analyses correct knowledge responses and positive attitude responses were coded as favorable, and practice questions were considered as favorable if the respondent reported “always” adhering to the given IPC practice (Supplementary Table 1). These were summarized both at the individual level and the HCW cadre level. Missing data or nonresponses were excluded. For each domain of KAP, a ranking of “good” was assigned if the respondent provided >75% favorable responses within a given category, “fair” if the respondent provided 50%–75% favorable responses, and “poor” if the respondent provided <50% favorable responses.

We conducted mixed effects logistic regression models, accounting for hospital clustering by including a random intercept for hospitals, to assess the relationship between demographics, attitude, and HCW cadre on knowledge, attitudes, and practices, separately. In each univariate model, predictors with a 2-sided *P* value <.25 were included in the multivariate analysis. We examined the multicollinearity of independent variables before including them in the final models. We calculated adjusted odds ratios (AORs) with 95% confidence intervals (CIs). All statistical analyses were performed using Stata 13 software (Stata Corp. College Station, TX).

## RESULTS

### General Characteristics of Participating Healthcare Workers

Of the 1794 HCWs approached, a total of 1728 were enrolled in the study, resulting in a response rate of 96.3%. The majority of participants were nurses (54.1%, n = 934), followed by physicians (30.4%, n = 526) and cleaning staff (15.5%, n = 268) (Table 1). The mean age of HCWs was 33.6 years (standard deviation ±8.9), and HCWs had a mean of 9.0 years (standard deviation ±8.5) of professional experience in the same role in healthcare. Nearly all the nurses (93.6%) were female, but more than half of both physicians and cleaning staff were male. In terms of educational attainment, all physicians and nurses held professional degrees (Bachelor of Medicine or Surgery or above, and Bachelor of Science or Diploma in nursing, respectively). Most of the cleaning staff (95.1%) had a secondary school certificate degree (10th grade) or below education.

**Table 1.** General Characteristics of Healthcare Workers in Selected Bangladesh Tertiary Care Hospitals, September 2020–January 2021

General Characteristics	Overall n = 1728	Physician n = 526	Nurse n = 934	Cleaning Staff n = 268
	n (%)			
Age				
Mean (± SD)	33.6 (±8.9)	31.1 (±7.2)	33.4 (±8.9)	39.0 (±9.9)
<25 y	205 (11.9)	65 (12.4)	121 (13.0)	19 (7.1)
25–40 y	1161 (67.2)	402 (76.4)	624 (66.8)	135 (50.4)
>40 y	362 (21.0)	59 (11.2)	189 (20.2)	114 (42.5)
Sex				
Female	1214 (70.2)	232 (44.1)	874 (93.6)	108 (40.3)
Male	514 (29.8)	294 (55.9)	60 (6.4)	160 (59.7)
Education				
Bachelor of Medicine/Surgery/doctorate/above	529 (30.6)	526 (100.0)	3 (0.3)	-
Bachelor of Science/Diploma in Nursing	931 (53.8)	-	931 (99.7)	-
Grade 12	13 (0.8)	-	-	13 (4.8)
≤Grade 10	255 (14.8)	-	-	255 (95.1)
Years of experience in the same healthcare field				
Mean ± SD	9.0 (±8.5)	6.0 (±6.6)	9.4 (±8.5)	13.9 (±9.2)
<5 y	699 (40.4)	270 (51.3)	373 (39.9)	56 (20.9)
5–15 y	632 (36.6)	205 (39.0)	349 (37.4)	78 (29.1)
>15 y	397 (23.0)	51 (9.7)	212 (22.7)	134 (50.0)

Abbreviation: SD, standard deviation.

### IPC Knowledge, Attitudes, and Practices Among HCWs

**Figure 1.**
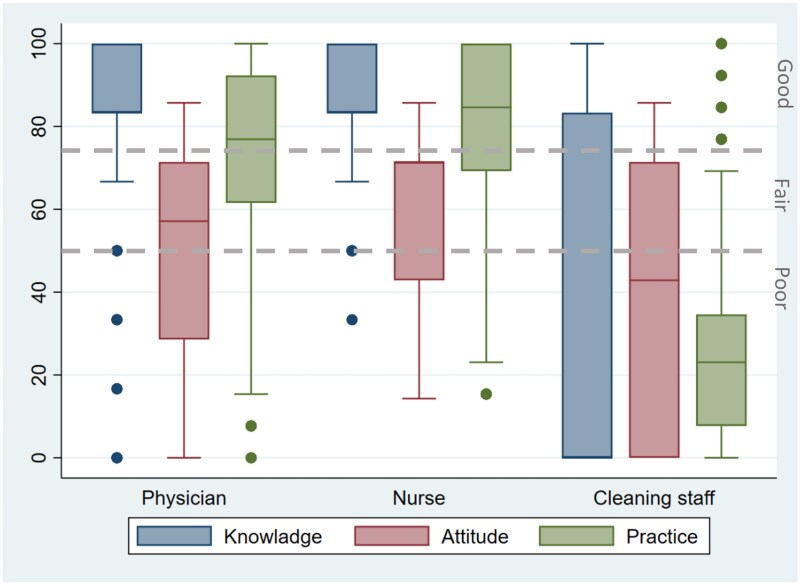
Median (interquartile range) score of knowledge, attitude, and practice level toward infection prevention and control among healthcare workers in selected Bangladesh tertiary care hospitals, September 2020–January 2021.

We found that IPC knowledge varied widely among HCWs (Figure 1, Supplementary Table 2). Specifically, physicians and nurses exhibited “good” levels of IPC knowledge with median scores of 94.8% and 96.6% favorable responses, respectively. However, IPC knowledge among cleaning staff ranked as “poor” with a median score of 47.3% favorable responses. Within the knowledge questions, physicians and nurses frequently answered questions about knowing hospital guidelines for HAI prevention, indications for hand hygiene, and standard precautions correctly (>90%), whereas they were less familiar with the need for isolation and cohorting in non-outbreak situations (Table 2).

**Table 2.** Knowledge, Attitude, and Practice Toward IPC Among Healthcare Workers in Selected Bangladesh Tertiary Care Hospitals, September 2020–January 2021^a^

Knowledge-related Question (Correct Response)	Overall	Physician	Nurse	Cleaning Staff
	% (n/N)			
I am familiar with hospital-acquired infection guidelines	91.1 (1530/1680)	94.6 (492/520)	98.1 (916/934)	54.0 (122/226)
I know the World Health Organization's (WHO) 5 moments of hand hygiene	89.5 (1500/1676)	95.2 (496/521)	97.3 (904/929)	44.2 (100/226)
Nosocomial infection is an infection that the patient brings from home^b^	65.7 (1086/1653)	79.9 (410/513)	71.7 (656/915)	8.9 (20/225)
Standard precautions apply to all patients regardless of their diagnosis	92.1 (1545/1678)	97.3 (507/521)	98.4 (917/932)	53.8 (121/225)
I am familiar with the isolation and cohorting of infectious patients	89.2 (1497/1678)	95.0 (495/521)	95.8 (895/934)	48.0 (107/223)
Isolation and cohorting are only needed in outbreak situations^b^	60.4 (1000/1656)	52.6 (273/519)	67.8 (631/931)	46.6 (96/206)
Attitude-related question (positive attitude)				
Policies and procedures for IPC should be adhered to at all times	98.1 (1636/1667)	99.0 (515/520)	99.5 (929/934)	90.1 (192/213)
I feel that the IPC policies and guidelines are sufficient in the hospital	67.4 (1119/1661)	59.1 (306/518)	73.4 (685/933)	61.0 (128/210)
I have enough time to comply with infection prevention guidelines	64.6 (1082/1674)	60.8 (317/521)	69.0 (644/934)	55.2 (121/219)
Resources are available to comply with IPC guidelines in this hospital	59.6 (990/1660)	51.5 (267/518)	64.1 (597/931)	59.7 (126/211)
Patients expect me to wash my hands before and after touching them	83.0 (1353/1630)	73.3 (378/516)	91.0 (849/933)	69.6 (126/181)
The workload affects my ability to follow infection prevention guidelines^b^	28.6 (479/1675)	32.4 (169/521)	27.2 (254/934)	25.4 (56/220)
Practice-related question (safe practice)				
I wash my hands before and after direct contact with the patient	57.4 (986/1717)	52.4 (272/519)	70.6 (657/931)	21.3 (57/267)
I wash my hands before and after wearing gloves	64.4 (1102/1710)	66.2 (343/518)	76.0 (703/925)	21.0 (56/252)
I wear gloves when performing invasive procedures	73.1 (1258/1721)	86.3 (453/525)	80.4 (746/928)	22.0 (59/268)
I attend in-service training/workshops related to infection control regularly	33.7 (530/1572)	33.5 (157/469)	41.7 (355/851)	7.1 (18/252)
I dispose of sharp instruments immediately after use	74.3 (1270/1709)	82.2 (424/516)	85.4 (790/925)	20.9 (56/268)
I keep/dispose of used gloves and other items in the proper place	80.6 (1385/1719)	87.7 (457/521)	92.2 (858/931)	26.2 (70/267)
I change gloves between patients irrespective of the patient's infectious status	65.3 (1103/1689)	65.5 (333/508)	80.0 (736/920)	13.0 (34/261)
I wear a gown/apron if soiling with blood or body fluids is likely	67.1 (1139/1698)	74.7 (383/513)	78.4 (721/920)	13.2 (35/265)
I wear a disposable facemask whenever there is the possibility of a splash or splatter	69.4 (1178/1698)	76.2 (388/509)	80.0 (738/923)	20.0 (52/266)
Staff/cleaners clean up hospital surface blood spills immediately using disinfectant	68.8 (1189/1727)	54.5 (287/526)	81.5 (762/934)	52.2 (140/268)
Do you use hand sanitizer?	81.7 (1412/1728)	77.6 (408/526)	86.6 (809/934)	72.8 (195/268)
I inform other healthcare personnel about infectious patients before the transfer of the patients	86.4 (1493/1728)	93.7 (493/526)	98.0 (915/934)	31.7 (85/268)
I inform authorities about patients with highly transmissible infections	88.5 (1530/1728)	94.7 (498/526)	97.6 (912/934)	44.8 (120/268)

Abbreviation: IPC, infection prevention and control.

^a^Denominator varied due to missing for nonresponse.

^b^Denotes response that was reverse scored.

The most frequent ranking among all categories of HCWs on attitudes toward IPC was “fair.” Nurses overall scored better on attitudes toward IPC with a median score of 71.2% favorable responses compared with physicians at 60.0% and cleaning staff at 60.4%. Among the attitude questions, consistently adhering to policies and procedures for IPC was found to have the most frequent favorable responses (90%–99% reporting a favorable response). The question with the lowest favorable response across all HCWs was regarding workload impacting the ability to follow national IPC guidelines (25%–32% reporting a favorable response) (ie, most HCWs think their workload negatively impacts their ability to follow IPC guidelines). More than one third of respondents indicated that IPC policies and guidelines in the hospital are not sufficient and that there is not enough time to comply with IPC guidelines. Almost half of respondents indicated that resources are not available to comply with IPC guidelines.

Physicians and nurses ranked as having “good” self-reported IPC practices. Fifty-two percent of physicians and 69% of nurses reported consistently adhering to IPC recommendations. Among the IPC practices, self-reported adherence by physicians and nurses was highest for informing hospital authorities and other healthcare workers about patients with transmissible infections (>90% reporting a favorable response). The lowest favorable responses were found for washing hands before and after direct contact with patients (52% of physicians and 71% of nurses reported a favorable response) and regularly attending in-service trainings or workshops related to infection control (34% of physicians and 42% of nurses reported a favorable response). The majority of cleaning staff ranked as having “poor” self-reported IPC practices with only 9% consistently adhering to IPC recommendations. Among the IPC practices, responses ranged from 7% reporting attending in-service training/workshops related to infection control to 73% reporting using hand sanitizer.

### Factors Associated With Good IPC Knowledge, Attitude, and Practice Among HCWs

Table 3 depicts factors associated with favorable IPC KAP categories in the multivariate analyses. Good IPC knowledge was associated with HCWs aged more than 40 years (AOR 2.3; 95% CI, 1.1–4.7). Conversely, HCWs with less than 15 years of working experience had almost twice the odds of having good IPC knowledge as those with more than 15 years of experience. HCWs with positive attitudes had 3 times the odds of having good knowledge of IPC (AOR 3.0; 95% CI, 1.8–4.9; *P* < .001) compared with those with poor IPC attitudes. Additionally, the odds of having a good level of IPC knowledge were higher among physicians (AOR 14.3; 95% CI, 9.3–21.9) and nurses (AOR 14.0; 95% CI, 9.2–21.2) compared to cleaning staff.

**Table 3.** Factors Associated With Good IPC Knowledge, Attitude, and Practices of IPC Among Healthcare Workers in Selected Bangladesh Tertiary Care Hospitals, September 2020–January 2021

Variables	HCWs (Physicians, Nurses, Cleaning Staff)					
	Good Knowledge		Good Attitude		Good Practice	
	AOR (95% CI)	*P* Value	AOR (95% CI)	*P* Value	AOR (95% CI)	*P* Value
Sex						
Men	Ref		Ref		Ref	
Women	1.3 (.9–1.8)	.130	0.9 (.6–1.3)	.561	1.6 (1.2–2.3)	.003
Age						
<25 y	Ref		Ref		Ref	
25–40 y	1.3 (.8–2.0)	.273	1. 0 (.6–1.6)	.925	0.8 (.5–1.2)	.208
>40 y	2.3 (1.1–4.7)	.028	0.9 (.4–2.0)	.721	0.7 (.3–1.3)	.252
Working experience						
>15 y	Ref		Ref		Ref	
<5 y	1.9 (1.1–3.5)	.034	1.2 (.6–2.5)	.661	0.7 (.4–1.3)	.312
5–15 y	1.8 (1.0–3.2)	.048	2.6 (1.3–5.3)	.008	1.2 (.7–2.1)	.604
Attitude						
Poor (<50)	Ref		N/A		Ref	
Fair (50–75)	1.6 (1.2–2.2)	.001	-		5.8 (4.5–7.5)	<.001
Good (>75)	3.0 (1.8–4.9)	<.001	-		16.3 (10.5–25.2)	<.001
HCW cadre						
Cleaning staff	Ref		Ref		Ref	
Physicians	14.3 (9.3–21.9)	<.001	4.2 (2.3–7.5)	<.001	12.9 (7.8–21.3)	<.001
Nurses	14.0 (9.2–21.2)	<.001	4.5 (2.4–8.3)	<.001	18.3 (11.0–30.4)	<.001

Abbreviations: AOR, adjusted odds ratio; CI, confidence interval; HCW, healthcare worker; IPC, infection prevention and control; N/A, not available; Ref., reference.

For good attitude as the outcome, those with 5–15 years of experience were more likely to have positive attitudes toward IPC (AOR 2.6; 95% CI, 1.3–5.3) than those with more than 15 years of experience. Similarly to IPC knowledge, the odds of having a positive attitude about IPC were higher among physicians (AOR 4.2; 95% CI, 2.3–7.5) and nurses (AOR 4.5; 95% CI, 2.4–8.3).

Women reported higher compliance with IPC practices compared to men (AOR 1.6; 95% CI, 1.2–2.3). Additionally, HCWs who had a positive attitude toward IPC had 16 times higher odds of reporting adherence to IPC practices (AOR 16.3; 95% CI, 10.5–25.2) compared to those with poor attitudes. As with knowledge and positive attitudes, in comparison to cleaning staff, nurses (AOR 18.3; 95% CI, 11.0–30.4) and physicians (AOR 12.9; 95% CI, 7.8–21.3) were also more likely to report performing good IPC practices.

## DISCUSSION

Our study investigated the IPC-related knowledge, attitudes, and practices among physicians, nurses, and cleaning staff in selected tertiary care hospitals in Bangladesh during the COVID-19 pandemic. In this study, we found that HCWs’ knowledge and attitudes about IPC parallel their IPC practices, demonstrating the importance of education and a positive culture around IPC to enhance safe practices. We also found significant disparities in IPC knowledge and attitudes by HCW role. This underscores the necessity of involving all HCWs in IPC training and capacity building.

The study revealed that most physicians and nurses had good IPC knowledge but the cleaning staff had very limited understanding of IPC. These findings are consistent with other studies conducted in similar settings that identified physicians and nurses had better IPC knowledge, whereas cleaning staff were found to have a low level of IPC knowledge [23–25]. Additionally, in 1 study focused on cleaners in Ethiopia, although overall IPC knowledge was poor, having good IPC knowledge correlated with good IPC practices, which, in turn, was associated with the availability of IPC guidelines [26]. Nurses performed better than physicians in many of the knowledge item questions, which is in line with a study conducted in Pakistan, where higher knowledge was noted among nurses compared to physicians [27]. One possibility for better knowledge among nurses is that nurses are more directly involved in patient care and therefore IPC is more relevant. However, cleaning staff also assist in patient care, though their role in patient care may be less well-recognized. This may result in them being excluded from IPC training. The latter was highlighted by the infrequent reports of attending in-service trainings for IPC among all HCWs, but especially cleaning staff. Moreover, differences in education may indicate that training materials need to be tailored to different cadres of HCWs, ensuring comprehensible language and appropriate visuals. Trainings should also be integrated into existing workflows to minimize disruptions. To ensure substantial IPC improvement, cleaning staff should be incorporated into the IPC program. HCWs were highly supportive of IPC policies and procedures, which may also be the reason for the high response rate, particularly with the attention given to IPC during COVID-19. However, several responses indicate administrative barriers to maintaining good IPC practices. For example, respondents frequently indicated that IPC policies and guidelines in the hospital are not sufficient, that there is not enough time to comply with IPC guidelines, and that resources are not available for complying with IPC guidelines. Moreover, the majority of participants indicated that their workload affects their ability to follow IPC guidelines. These concerning trends demonstrate that some of the major obstacles to IPC improvements may depend on administrative changes such as increasing the frequency of IPC workshops, hiring additional staff to reduce workloads, and ensuring consistent availability of PPE to support IPC activities. These findings align with other studies that have found staffing ratios to negatively impact IPC [28, 29]. Although some of these findings may have been exacerbated because of the influx of patients during the COVID-19 pandemic, other reports have indicated that these problems preceded COVID-19 [30]. Regardless, strategies are needed to accommodate patient surges to ensure IPC can be maintained as IPC is even more critical in the setting of infectious disease outbreaks where healthcare facilities can otherwise become hot spots for disease spread.

Self-reported IPC practices were generally good among nurses and physicians, though responses to individual questions demonstrate areas for improvement. Notably, washing hands before and after patient contact and changing gloves between patients were not universally performed by nearly one third of nurses and physicians. As noted previously, this lack of compliance may be related to resource limitations (particularly hand washing agents and appropriate PPE) and time constraints (high patient workloads, limited staffing) that many participants acknowledged. Similar findings were reflected in studies conducted in Nigeria, Bangladesh, and Pakistan where resource constraints hindered IPC practices [31–34]. A more substantial deficit was found among the practices of cleaning staff where consistent adherence to most IPC practices was reported by less than 10% of participants. Cleaning staff are a crucial component of hospital services. However, their KAP scores are likely significantly impacted by low literacy rates, limited access to training opportunities, and insufficient empowerment within the hospital hierarchy. Addressing these issues is essential for enhancing their effectiveness and improving overall hospital IPC. This finding is also consistent with a study recently conducted in a similar setting [26, 33]. This again indicates the pressing need to provide repeated training and sensitization to cleaning staff in addition to administrative support as part of a comprehensive IPC program. Additionally, tailored training programs focused on visual aids, hands-on demonstrations, and role-playing would likely have greater impact in the setting of low literacy. Moreover, training should be implemented alongside routine or periodic monitoring and feedback mechanisms to encourage continuous improvement. Ongoing assessments are needed to identify gaps in existing IPC infrastructure and PPE supplies and involve hospital authorities in the appropriate mobilization of resources, particularly as resource needs change, such as during an outbreak.

Good knowledge of IPC was found to be positively associated with increasing age, though it was negatively associated with years of experience. It is unclear why these 2 variables would be inversely related, though perhaps more recent education or training through staff orientation may have enhanced knowledge among recently hired staff. One of the key findings was the strong association between a positive attitude toward IPC and self-reported adherence to IPC practices. This finding is supported by a systematic review that reported similar findings [35]. A possible explanation might be that HCWs who felt that compliance with standard precautions would protect them against HAIs were more inclined toward safe IPC practices. Alternatively, HCWs who felt supported and equipped to perform IPC activities may have been more likely to adhere to IPC. Regardless, hospitals should foster a positive IPC environment by setting IPC as a priority and providing staff with the resources they need to ensure the implementation of IPC guidance. Additionally, cleaning staff should be empowered to speak up about potential infection risks, creating a culture of safety where they feel comfortable raising concerns without fear of retaliation.

The interdependence of KAP components in supporting IPC has been previously reported in the literature. A study conducted in Nigeria revealed that although HCWs were well-informed about the transmission of HAIs, their compliance with standard precautions was inadequate [31]. This suggests that knowledge alone is not enough to change HCW behavior; improvements in IPC attitudes and practices are also vital to IPC compliance [36].

There are some limitations in our study design. Because we enrolled participants mostly from public tertiary care hospitals, the sample was not representative of the HCW population overall, and therefore the results may not be generalizable across all healthcare facilities. To reduce the burden on HCWs during the COVID-19 response, a small number of items were selected to measure each domain, which may have limited our ability to understand the depth and breadth of IPC KAP. Additional in-depth interviews or surveys, especially with cleaning staff, could yield additional insights. Because of the cross-sectional study design, we could not show any temporality between different covariates and IPC compliance. KAP responses were self-reported and may overestimate actual compliance with IPC practices. Response bias may have influenced physicians and nurses when answering questions about policy awareness as they might have felt compelled to report familiarity even if it was lacking. This may be less of an issue for cleaners who may not feel responsible for knowing those guidelines or not feel empowered to know them. Finally, this study was conducted during the COVID-19 pandemic and may not wholly reflect practices before or subsequent to the outbreak. Future research endeavors are advised to examine the evolution of IPC practices in non-pandemic circumstances. Such investigations will contribute to a more comprehensive understanding of standard infection control practices.

In light of our findings, several specific IPC knowledge and practice gaps have been identified that need to be addressed in future infection control programs to ensure resiliency beyond the COVID-19 pandemic. Cleaning staff were found to lag behind in all aspects of IPC, likely arising from a shortage of designated training and job orientation. Training materials tailored for cleaning staff, including those with low or no literacy, will make it easier for them to become acquainted with the concepts and importance of IPC. Additionally, the correlation between a positive attitude toward IPC with improved IPC practices demonstrates the interdependency of the various KAP components. However, even the most positive attitude toward IPC will not translate into safe practices if there is not a facilitating environment. Therefore, hospital leadership needs to be engaged to create a positive culture around IPC, which will depend on adequate training and monitoring, appropriate staffing, provision of IPC materials, and role modeling by senior leaders. Policymakers and hospital administrators should implement practical steps like establishing hospital-wide IPC committees that include representatives from all healthcare cadres, including cleaning staff. Closing IPC gaps is a critical component of reducing HAIs and is an ethical imperative to improve safety of healthcare settings.

## Supplementary Material

ciaf246_Supplementary_Data

## Supplementary Data

Supplementary materials are available at *Clinical Infectious Diseases* online. Consisting of data provided by the authors to benefit the reader, the posted materials are not copyedited and are the sole responsibility of the authors, so questions or comments should be addressed to the corresponding author.

## Notes

***Acknowledgments.*** The authors sincerely thank all the study participants and project hospitals for their valuable time and support. Furthermore, the authors gratefully acknowledge the utmost support contributed by the US Centers for Disease Control and Prevention (US-CDC, Grant # 6NU51GH001209) and DGHS for their continuous dedication and efforts to promote novel approaches to infection prevention and control. icddr,b is also grateful to the governments of Bangladesh and Canada for providing core/nrestricted support.

***Author Contributions.*** M. G. D. H., M. M. U. A., S. A. S., M. S. I., A. R. S., L. P. O., P. A. M., and S. C. K. D. led the conceptualization, data curation, methodology, analysis, and writing of the paper. M. G. D. H., M. M. U. A., A. R., M. S. I., S. A. S., and A. R. S. led the design of the study. M. G. D. H., S. A. H. M. A., and T. M. M. contributed to data collection and curation. M. G. D. H., M. M. U. A., S. A. S., S. A. H. M. A., A. R., M. S. I., A. R. S., L. P. O., P. A. M., and S. C. K. D. critically reviewed and revised the article for content and language. All authors approved the final version of the manuscript.

***Financial support**.* This work was supported by the Centers for Disease Control and Prevention (CDC). Award No-6NU51GH001209 September 2018–September 2020.

***Ethical approval.*** We conducted the study after the protocol was reviewed and approved by the Research Review Committee and Ethical Review Committee under the institutional review board of icddr,b. We obtained permission from the hospital administration of the study sites. We obtained written informed consent from all participants. Participants were provided with anonymous ID numbers as anonymity and confidentiality was strictly maintained.

***Potential conflicts of interest**.* The authors: No reported conflicts of interest. All authors have submitted the ICMJE Form for Disclosure of Potential Conflicts of Interest.
