# Supplementary material for: Infection Prevention and Control Knowledge, Attitudes, and Practices of Healthcare Workers in Tertiary Care Hospitals in Bangladesh During COVID-19: A Multicenter Cross-sectional Survey
Source: Clin Infect Dis. 2025 Jun 25;81(1):49–56. doi: 10.1093/cid/ciaf246 (PMC12314486; doi:10.1093/cid/ciaf246)
Supplement: ciaf246_Supplementary_Data [file ciaf246_supplementary_data.zip › Supplement Table 1 and 2_125252.docx]

**Supplement Table 1. Assignment of knowledge, attitude, and practice ranking categories for measures of IPC among healthcare workers**

| **KAP Ranking** | |
| --- | --- |
| **Knowledge** |  |
| Good | >75% correct responses |
| Fair | 50-75% correct responses |
| Poor | <50% correct responses |
| **Attitude** |  |
| Good | >75% positive responses |
| Fair | 50-75% positive responses |
| Poor | <50% positive responses |
| **Practice** |  |
| Good | >75% of IPC practices reported as “always” adhered to |
| Fair | 50-75% of IPC practices reported as “always” adhered to |
| Poor | <50% of IPC practices reported as “always” adhered to |

**Supplement Table 2: Knowledge, attitude, and practice level toward IPC among healthcare workers in selected Bangladesh tertiary care hospitals, September 2020 to January 2021**

| **Categories of KAP** | **Overall**  **n=1728** | **Physician n=526** | **Nurse**  **n=934** | **Cleaning Staff n=268** |
| --- | --- | --- | --- | --- |
|  |  | **n (%)** |  |  |
| **Knowledge** |  |  |  |  |
| Good | 1328 (76.8) | 440 (83.6) | 807 (86.4) | 81 (30.2) |
| Fair | 190 (11.0) | 62 (11.8) | 106 (11.4) | 22 (8.2) |
| Poor | 210 (12.2) | 24 (4.6) | 21 (2.2) | 165 (61.6) |
| **Attitude** |  |  |  |  |
| Good | 279 (16.2) | 98 (18.6) | 165 (17.7) | 16 (5.9) |
| Fair | 788 (45.6) | 184 (35.0) | 494 (52.9) | 110 (41.1) |
| Poor | 661 (38.2) | 244 (46.4) | 275 (29.4) | 142 (53.0) |
| **Practice** |  |  |  |  |
| Good | 944 (54.6) | 272 (51.7) | 647 (69.3) | 25 (9.3) |
| Fair | 382 (22.1) | 162 (30.8) | 195 (20.9) | 25 (9.3) |
| Poor | 402 (23.3) | 92 (17.5) | 92 (9.8) | 218 (81.4) |

Good: >75% favorable responses; Fair: 50-75% favorable responses; Poor: <50% favorable responses
